# 3D play: from bench to hybrid catheterization laboratory

**DOI:** 10.1093/ehjimp/qyaf034

**Published:** 2025-03-20

**Authors:** Simona Celi, Katia Capellini, Benigno Marco Fanni, Alessandra Pizzuto, Giuseppe Santoro, Emanuele Gasparotti

**Affiliations:** BioCardioLab, Bioengineering Unit, Fondazione Toscana G. Monasterio, Massa, Italy; BioCardioLab, Bioengineering Unit, Fondazione Toscana G. Monasterio, Massa, Italy; BioCardioLab, Bioengineering Unit, Fondazione Toscana G. Monasterio, Massa, Italy; Pediatric Cardiology and GUCH Unit, Fondazione Toscana G. Monasterio, Massa, Italy; Pediatric Cardiology and GUCH Unit, Fondazione Toscana G. Monasterio, Massa, Italy; BioCardioLab, Bioengineering Unit, Fondazione Toscana G. Monasterio, Massa, Italy

The integration of engineering and cardiology is revolutionizing interventional planning through the use of 3D modelling and printing. By leveraging advanced tomographic imaging techniques such as computed tomography (CT) and magnetic resonance imaging (MRI), patient-specific 3D models of cardiovascular structures can be generated and utilized to optimize interventional procedures.^1,2^

*3D model creation and printing techniques:* The process begins with the acquisition of high-resolution CT or MRI scans (*Figure 1A*), which are then processed using specialized segmentation software able to convert the DICOM (Digital Imaging and COmmunications in Medicine) medical images into a detailed 3D digital reconstruction of the target anatomy. Once the digital model is refined, it is exported in a STL format and prepared for 3D printing (*Figure 1B*). Various printing technologies can be employed based on the clinical needs, including stereolithography for high precision, fused deposition modelling for cost-effective solutions, and selective laser sintering for durable and flexible models.^3^

**Figure 1:**
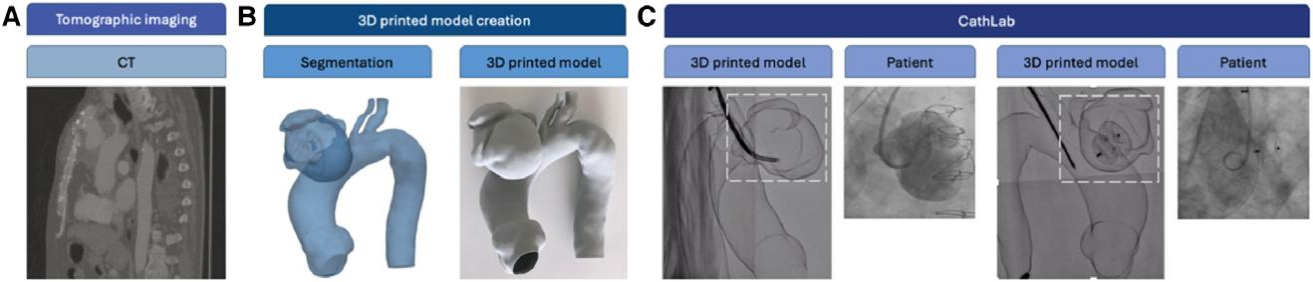
Example of creation and application of a 3D printed model from bench to cath lab: CT image (*A*), 3D printing model creation (*B*), and two different phases of the device deployment in the printed model in the CathLab (*C*).

The rationale behind adopting these various techniques is that each printing method is tailored to a specific set of materials and operates with different parameters, such as printing thicknesses and the feasibility of creating complex structures. More recently, silicone printing technologies by direct or indirect printing are also available. These processes, although more complex, allow the advantages of the material's transparency and flexibility to be exploited.^4^ The choice of material and printing method is tailored to replicate the overall mechanical properties of the target structures, ensuring accurate simulation of anatomical features and interventional conditions. The combination of different Young’s moduli of the printed material and of wall thickness of the printed structure allows for a wide range of overall stiffness of the printed model.^5^

*Application in the hybrid cath lab:* Once printed, the 3D model is introduced into the catheterization laboratory (cath lab) to serve as a pre-procedural planning tool (*Figure 1C*). The interventional team can use the model to simulate the procedure, evaluate device positioning, and anticipate anatomical challenges. By mimicking the patient’s anatomy, the model enables operators to test different approaches, select the optimal interventional strategy, and refine procedural techniques in a risk-free environment. During fluoroscopic evaluation, the model is placed in the cath lab to simulate the procedural steps. By introducing interventional devices such as catheters, stents, and guidewires into the 3D model under fluoroscopy, clinicians can assess vascular access points, device manoeuvrability, and optimal deployment strategies. This simulation allows for the following:

- Evaluation of different intervention techniques, including transcatheter valve implantation, percutaneous coronary interventions, and structural heart procedures.
- Fluoroscopic analysis of device behaviour, ensuring accurate positioning and reducing the need for intraoperative trial and error.
- Hands-on team training and coordination, improving communication between operators and refining workflow dynamics before the actual procedure.
- Assessment of procedural risks, including vascular complications, access difficulties, and device–host interactions.

*Clinical and economic benefits:* This practice has demonstrated several advantages:

- Optimization of procedural planning, leading to improved accuracy and reduced complications.
- Reduction of radiation exposure for both patients and operators, as fluoroscopic time can be minimized.
- Decreased procedure time by allowing clinicians to foresee and address anatomical challenges before the intervention.
- Lowered costs associated with shorter procedural durations, reduced anaesthesia use, and improved resource allocation.

*Conclusion:* The integration of 3D modelling into cardiovascular interventions represents a paradigm shift in procedural planning. This interdisciplinary approach enhances precision, improves safety, and contributes to better patient outcomes. Future research should focus on standardizing 3D model applications and assessing their long-term clinical and economic impact.

## Data Availability

No new data were generated or analysed in support of this research.

## Lead author biography

Dr Simona Celi is an engineer. She is the Director of the Bioengineering Unit at Fondazione Toscana G. Monasterio, Massa and Pisa, Italy. Dr Celi has deep experience in bioengineering research on cardiovascular diseases combining numerical, experimental, and translational approaches. She has contributed to several international high-profile projects.
